# Cell Death Signaling From Endoplasmic Reticulum Stress: Plant-Specific and Conserved Features

**DOI:** 10.3389/fpls.2022.835738

**Published:** 2022-02-03

**Authors:** Eduardo B. Simoni, Célio C. Oliveira, Otto T. Fraga, Pedro A. B. Reis, Elizabeth P. B. Fontes

**Affiliations:** Department of Biochemistry and Molecular Biology, BIOAGRO, National Institute of Science and Technology in Plant-Pest Interactions, Universidade Federal de Viçosa, Viçosa, Brazil

**Keywords:** endoplasmic reticulum, cell death signaling, autophagy, plant immunity, ER stress, programmed cell death, unfolded protein response

## Abstract

The endoplasmic reticulum (ER) stress response is triggered by any condition that disrupts protein folding and promotes the accumulation of unfolded proteins in the lumen of the organelle. In eukaryotic cells, the evolutionarily conserved unfolded protein response is activated to clear unfolded proteins and restore ER homeostasis. The recovery from ER stress is accomplished by decreasing protein translation and loading into the organelle, increasing the ER protein processing capacity and ER-associated protein degradation activity. However, if the ER stress persists and cannot be reversed, the chronically prolonged stress leads to cellular dysfunction that activates cell death signaling as an ultimate attempt to survive. Accumulating evidence implicates ER stress-induced cell death signaling pathways as significant contributors for stress adaptation in plants, making modulators of ER stress pathways potentially attractive targets for stress tolerance engineering. Here, we summarize recent advances in understanding plant-specific molecular mechanisms that elicit cell death signaling from ER stress. We also highlight the conserved features of ER stress-induced cell death signaling in plants shared by eukaryotic cells.

## Introduction

The endoplasmic reticulum (ER) is the gateway of synthesized proteins by ER membrane-bound polysomes to the secretory pathway. It is a multitask intracellular organelle that provides the functional apparatus for translocation of the newly synthesized secretory proteins to the lumen of the organelle, protein folding, and protein post-translational modifications. These protein processing activities allow nascent proteins to their destination in the secretory pathway. Under normal conditions, the rate of protein processing in the ER lumen is balanced with the protein synthesis rate and loading into the organelle. Stress conditions that disturb this equilibrium and promote the accumulation of unprocessed, misfolded protein in the organelle promote ER disfunction, a process known as ER stress. To minimize the deleterious effect of misfolded proteins and prevent their translocation further in the secretory pathway, a protein quality control machinery monitors protein folding. It addresses misfolded proteins to degradation *via* either the ER-associated degradation (ERAD) system or autophagy. The perturbations in the ER function activate signaling cascades that allow ER communication with the cytoplasm, nucleus, and, under chronically prolonged ER stress, mitochondria, and vacuole to restore ER homeostasis or ultimately cause programmed cell death (PCD).

The unfolded protein response (UPR) is an evolutionarily conserved signaling pathway activated in response to ER stress. Plant UPR is transduced as a well-characterized bipartite signaling module consisting of the ER membrane-associated transducers inositol-requiring protein 1 (IRE1) and bZIP (basic leucine zipper) transmembrane transactivation factors. In Arabidopsis, two copies of IRE1, *Arabidopsis thaliana* (At)IRE1a and AtIRE1b, and two copies of the transmembrane bZIP, AtbZIP28, and AtbZIP17, with partially overlapping functions operate in UPR. The functional conservation of these UPR transducers has been examined in other plant species and extends to include eukaryotes from other kingdoms. Under physiological conditions, the luminal domain of AtbZIP28 is bound to the ER-resident molecular chaperone binding protein (BiP) that prevents its activation. Under ER stress conditions, the demand for the chaperone function of BiP is increased, then BiP dissociates from bZIP28, allowing its translocation to the Golgi where it is proteolytically processed to release the bZIP domain from the membrane and promote its translocation to the nucleus. ER stress also activates the kinase and endonuclease domains of the second UPR transducer IRE1, which promotes unconventional splicing of AtbZIP60 RNA to delete a transmembrane motif-encoding segment of the AtbZIP60u unspliced RNA. The IRE-mediated unconventional splicing results in the translation of AtbZIP60s spliced RNA into a soluble transactivation factor that is translocated to the nucleus. AtbZIP60 is the primary downstream component of the IRE1 signaling module, which acts in concert with AtbZIP28 to induce the expression of ER protein processing-related genes involved in the ER protein folding machinery and PCD system. Furthermore, the nuclease activity of IRE1 degrades mRNA encoding secretory proteins, a process known as Regulated IRE1-Dependent RNA Decay (RIDD), to reduce protein loading into the lumen, thereby decreasing protein folding demands within the organelle. However, extensive and acute ER stress directs the UPR toward activating cell death-triggering pathways.

This review describes recent advances in understanding the molecular mechanisms underlying the ER stress responses in plants. It focuses primarily on (a) plant UPR and their connections with cell death mechanisms; (b) ER stress-induced plant-specific cell death signaling; (c) ER stress-mediated autophagy; and (d) ER stress-induced PCD in plant immunity.

## Plant ER Stress Elicitation and Conserved Features of Plant UPR

The precise operation of the ER is essential to maintaining cellular homeostasis as the ER is involved in several crucial processes, such as protein folding and maturation. Protein processing can fail even under normal conditions, leading to misfolded/unfolded proteins. To minimize the accumulation of unfolded proteins, two systems play essential roles, the ER quality control (ERQC) system and the ERAD system (reviewed in Liu and Howell, 2016). Nevertheless, under adverse environmental conditions or conditions of intense protein secretion, the demand for protein folding can exceed the efficiency of the folding and degradation systems, thereby, the cells might accumulate misfolded proteins in the ER lumen, leading the ER stress conditions (Liu and Howell, 2016).

Different biotic and abiotic stresses have been shown capable of causing ER stress. In the plant cells, ER stress can be induced by adverse environmental conditions, such as heat, salt, and drought (Liu et al., 2007b; Deng et al., 2011; Parra-Rojas et al., 2015). Similarly, pathogen diseases can also trigger an imbalance in ER functioning, leading to ER stress. Moreno et al. (2012), Ye et al. (2013), and Park and Park (2019). Furthermore, studies have shown that the plant hormones salicylic acid (SA) and ABA may be associated with ER stress (Yang et al., 2013; Nagashima et al., 2014; Zhou et al., 2015). In addition, certain chemical compounds, including tunicamycin (TM), dithiothreitol (DTT), and l-azetidine-2-carboxylic acid (AZC), can trigger ER stress. While TM prevents N-linked glycosylation of secreted glycoproteins, DTT interferes with the formation of disulfide bonds and, as an inhibitor of the ER calcium pump, AZC affects the primary components of the ER protein-folding apparatus, calnexin, and calreticulin, which are calcium-dependent, thereby, all of them are capable of disrupting the correct folding of proteins (Nawkar et al., 2018). How all elicitors work is not fully understood, but presumably, they may hinder the ER function in some way to indirectly affect protein folding (Howell, 2017).

The accumulation of misfolded proteins in the ER lumen establishes the condition known as ER stress that stimulates UPR. UPR is a conserved cytoprotective signaling pathway among eukaryotes (Wan and Jiang, 2016). This pathway is activated primarily to restore ER homeostasis through (i) an increase in ER chaperone synthesis for protein folding; (ii) upregulation of lipid synthesis to expand ER capacity; (iii) repression of global translation to control protein loading into the organelle; and (iv) upregulation of ERAD genes to attenuate unfolded protein accumulation in the ER lumen (Nawkar et al., 2018; Pastor-Cantizano et al., 2020).

**Figure 1:**
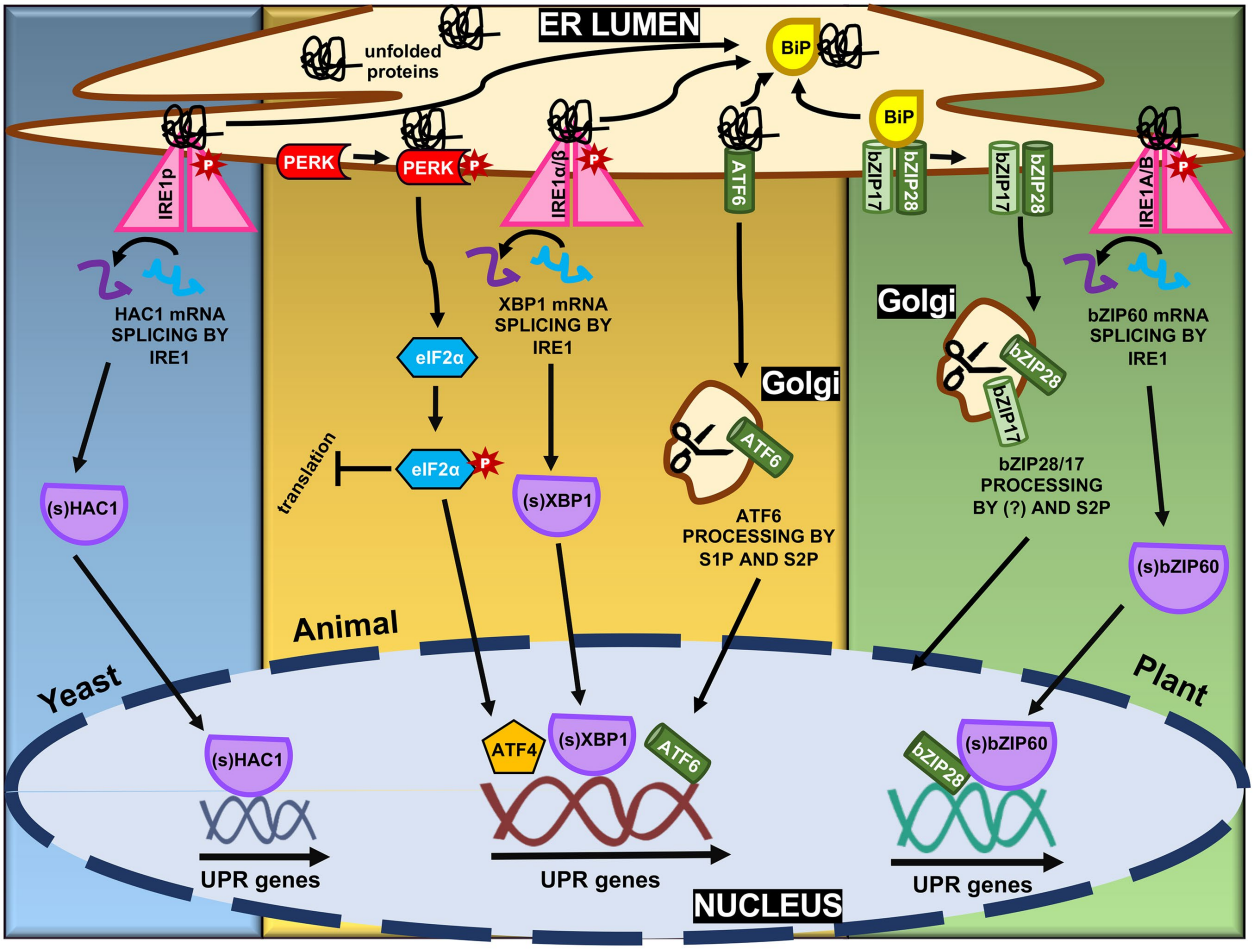
Molecular mechanisms of ER stress-induced unfolded protein response (UPR) signaling in yeast, animal, and plant. The ER stress transducers, Ire1α/β, PERK, and activating transcription factor 6 (ATF6) form the three branches of the UPR pathways in mammals. PERK oligomerizes and phosphorylates eIF2α to decrease overall translation while increasing specific translation of genes, including ATF4. Upon ER stress, ATF6-BiP complex dissociates and ATF6 is packaged and translocated to Golgi apparatus, where it is processed to create an active transcription factor. Ire1α/β, releasing from BiP and sensing misfolded proteins, oligomerizes and phosphorylates itself, leading to the activation of the XBP1 transcription factor by splicing the XBP1u mRNA to create XBP1s mRNA. All three transcription factors lead to the upregulation of UPR genes. In plants, with similar mechanisms, only Ire1α/β and ATF6 branches are identified, Ire1a/b and bZIP17/28, respectively. Yeast have only the Ire1α/β branch, represented by Ire1p.

**Figure 2:**
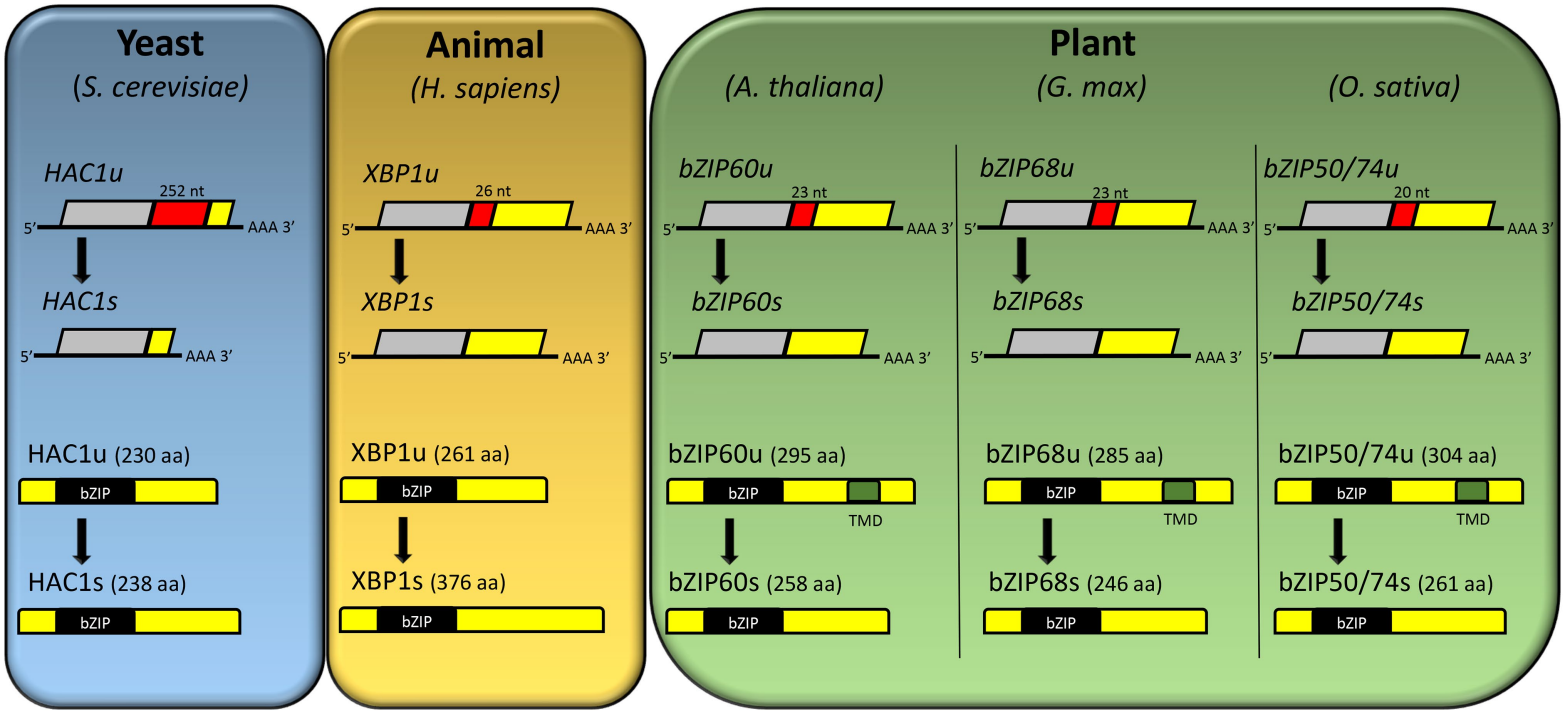
Comparison of IRE1-mediated unconventional splicing and resulting open reading frame (ORF) of *HAC1*, *XBP1*, *AtbZIP60*, *GmbZIP68*, and *OsbZIP74* (also known as *OsbZIP50*) mRNA. In yeast, a stop codon in *HAC1u* is removed by unconventional splicing, and the resulting *HAC1* mRNA (*HAC1s*) encodes the HAC1s protein. In animal, the unconventional splicing of *XBP1u* mRNA results in fusion of two ORFs in *XBP1s* mRNA, which then encodes a larger protein, XBP1s. In contrast to HAC1 and XBP1, the unconventional splicing of plant bZIPs mRNA (*bZIP60u*, *bZIP68u*, *bZIP50/74u*) produces a smaller protein (bZIP60s, bZIP68s, bZIP50/74s). The spliced sequences are highlighted in red with the number of spliced nucleotides. The transmembrane domains are highlighted in green. Adapted from Iwata and Koizumi (2012).

ER stress perception and, subsequently, UPR activation are mediated by membrane-associated sensors first identified in yeast and mammals (Fu and Gao, 2014). In yeast, the UPR is regulated by the inositol-requiring transmembrane kinase/endonuclease p, a type I transmembrane ER protein that removes an intron of 252 nucleotides from HAC1 mRNA, forming the mature mRNA Hac1p (Figure 1, Sidrauski and Walter, 1997; Maldonado-Bonilla, 2020). Hac1p encodes a transcription factor of 238 amino acids (Figure 2) that plays an essential role in the UPR signaling, regulating downstream UPR genes including *KAR2*, *PDI1*, *EUG1*, and *LHS1* (Xia, 2019). In metazoans, the UPR signaling pathway is modulated by three ER stress-sensing and transducing proteins (Figure 1). One branch of UPR signaling involves the bifunctional kinase and endoribonuclease IRE 1 (α and β subunits), which splices the mRNA of bZIP-like transcription factor X box-binding protein-1 (XBP1; Figure 2). A second branch is mediated by membrane-tethered activating transcription factor 6 (ATF6), transported to the Golgi to be processed by site 1 and site 2 proteases (S1P and S2P). In the third signaling branch, the global translation is regulated by ER-associated kinase R-like endoplasmic reticulum kinase (PERK), which phosphorylates and inactivates the translation initiation factor eIF2a (Wan and Jiang, 2016; Ruberti et al., 2018). These ER sensors are regulated by the ER-resident molecular chaperone BiP. Under normal conditions, BiP associates with the luminal portion of these receptors, keeping them inactive. Accumulation of misfolded proteins in the ER causes BiP to dissociate from these transducers to serve as a molecular chaperone. The BiP release promotes the activation of these receptors (Pincus et al., 2010; Srivastava et al., 2013; Li et al., 2017).

Plant functional homologs of ATF6 (bZIP17 and bZIP28) and IRE1 (IRE1a and IRE1b), but not PERK, have been described (Ruberti and Brandizzi, 2014). bZIP17 and bZIP28 are type II transmembrane proteins with a cytosolic N-terminal portion containing the bZIP transcription factor (TF) domain and an ER luminal C-terminus with amino acid signals for ER retention (Figure 1; Liu et al., 2007a). In non-stressed cells, BiP interacts with the luminal domain of bZIP17/28, keeping them retained in the ER (Figure 1). Upon ER stress, however, BiP disconnects from bZIP17/28 to act on misfolded proteins and, thus, allowing the translocation of these TFs from ER toward the Golgi complex (Srivastava et al., 2013). Once on the Golgi, an unidentified protease(s) first cleaves the bZIP28 at its transmembrane domain, thereby allowing S2P to function. Although the luminal portion has the consensus S1P recognition motif, S1P is not involved in bZIP28 processing (Iwata et al., 2017). In contrast, bZIP17 appears to suffer the action of S1P (Liu et al., 2007b). Once cleaved, the TF can reallocate to the nucleus forming a transcriptional complex with the nuclear factor-Y TFs to activate the UPR genes (Liu and Howell, 2010). In most cases, bZIP17 and bZIP28 exhibit similar properties and are induced by ER stress inducers (TM, DTT), environmental and developmental conditions. However, the induction kinetics, specific activation, and modulated target genes are not necessarily identical (Liu et al., 2007a,b; Duwi Fanata et al., 2013; Henriquez-Valencia et al., 2015; Li et al., 2017; Park and Park, 2019). For instance, bZIP17, but not bZIP28, is induced, processed, and relocated into the nucleus upon salt stress conditions. Kim et al. (2018) showed that the double mutant *bzip17/28* exhibited root growth impairment and constitutive overexpression of *the bZIP60* gene. These results indicate that either bZIP17 and bZIP28 function redundantly or act together to modulate cell growth and root development, besides the UPR activity (Kim et al., 2018). Likewise, bZIP17 functions in concert with IRE1a and IRE1b in normal plant development as the *bzip17/ire1a/ire1b* triple mutant displays severe vegetative and reproductive growth defects (Bao et al., 2018). IRE’s role in normal plant development was associated with IRE RIDD activity on secretory protein mRNAs but uncoupled to the bZIP60 mRNA splicing, whereas bZIP17 might activate UPR unrelated genes in response to developmental stimuli. Moreover, both bZIP17 and bZIP28 genes, along with the IRE/bZIP60 signaling arm, are induced by virus infection in Arabidopsis and *Nicotiana benthamiana* (Gayral et al., 2020; Li and Howell, 2021).

The second branch of plant UPR relies on the ER receptor IRE1 (Figure 1). In many eukaryotes, IRE1-mediated unconventional splicing of mRNA is the most conserved branch of the UPR (Ruberti et al., 2015). Like in metazoan, plant IRE1 is an ER membrane protein with dual functions. As an endoribonuclease, it catalyzes mRNA splicing; as a serine/threonine-protein kinase, IRE1 initiates autophosphorylation, forming oligomers upon ER stress (Wan and Jiang, 2016). As a central player in the UPR, IRE1 mediates two signaling pathways, the unconventional splicing of its typical substrate, bZIP60, and an alternative pathway that cleaves other RNAs by RIDD to control translational overload (Hollien et al., 2009; Mishiba et al., 2013). The IRE1 receptor configuration harbors an N-terminal signal-sensing portion facing the ER lumen, followed by an internal transmembrane segment and a cytosolic signal-transducing C-terminal domain. The N-terminal region senses misfolded proteins to trigger signaling. The C-terminal region plays a role as an RNA processing enzyme, acting at the unconventional splicing of bZIP60 transcription factor, another essential regulator of the ER stress response (Sidrauski and Walter, 1997; Deng et al., 2011; Wakasa et al., 2012). Although well-characterized in yeast, the exact mechanism of the IRE1 stress-sensing process in plants has not been elucidated yet (Zhang et al., 2016). The most accepted hypothesis is that similarly to bZIP17/28, BiP detaches from the luminal portion of the IRE1, and once released, this region interacts with misfolded proteins, activating the pathway (Kimata et al., 2003; Gardner and Walter, 2011; Li and Howell, 2021). Upon ligand-induced activation, IRE1 undergoes dimerization and autophosphorylation followed by oligomerization and cluster formation (Wan and Jiang, 2016; Nawkar et al., 2018). The RNase activity of IRE1 processes the mRNA encoding unspliced bZIP60 (bZIP60u) to produce an active TF, spliced bZIP60 (bZIP60s), which has 23 bp less (Figure 2; Howell, 2013). The two ends of bZIP60 mRNA in Arabidopsis are joined by a tRNA ligase RLG1 (Nagashima et al., 2016). Likewise, this process is conserved in soybean and rice, in which GmbZIP68 and OsbZIP50/74 mRNAs are processed upon ER stress induction (Figure 2). The active form of bZIP60 without the transmembrane domain translocates toward the nucleus and modulates the expression of UPR target genes to overcome ER stress (Deng et al., 2011). The Arabidopsis bZIP60 splicing is induced by a variety of conditions that cause the ER stress and, unlike other eukaryotes, the spliced and unspliced forms are present in any stressful condition; the extent of this processing is affected by the organ nature (Parra-Rojas et al., 2015). Moreover, bZIP60u protein has been shown to undergo constant action by the proteasome system, and its function remains elusive.

Among plant species, IRE1 may be represented by one or more isoforms depending on the species; for example, the rice genome encodes a single *IRE1* isoform, while Arabidopsis and soybean genomes encode two and four isoforms, respectively (Nagashima et al., 2011; Wakasa et al., 2012; Silva et al., 2015; Howell, 2021). Besides the two full-length isoforms, the Arabidopsis genome also has a third *IRE1* gene (*IRE1c*), which generates a truncated protein lacking the sensor domain and might play a role beyond the ER stress responses. This interpretation is supported by the finding that *IRE1c*, together with *IRE1a* and *IRE1b*, is essential for plant development since the triple knockout mutant *ire1a/b/c* is lethal (Mishiba et al., 2019). However, introducing a heterozygous IREc allele into the triple mutant generated the ire1a/ire1b/ire1c (−/+) mutant and caused a typical phenotype, linking IRE1c to male gametogenesis (Pu et al., 2019).

A specific and alternative plant UPR branch involves two NAC proteins that also require the bZIP60 function (Figure 1). The bZIP60-ANAC062/ANAC103 module is activated to amplify the transcriptional signals that ensure cell survival under ER stress conditions. Under accumulation of misfolded proteins into the ER lumen, the transmembrane segment-less bZIP60 is translated from the processed bZIP60 mRNA and directed to the nucleus, where it binds to the cis-element UPRE III on the ANAC062 and ANAC103 promoter region and induces the expression of these target genes (Sun et al., 2013; Yang et al., 2014a). The plasma membrane-associated transcription factor ANAC062 has a transmembrane domain, processed under ER stress, leading to the ANAC062 relocation from the plasma membrane to the nucleus (Yang et al., 2014a). Besides protein synthesis and traffic, the endoplasmic reticulum produces lipids and sterols, crucial for ER and plasma membrane biogenesis. Possibly, the ER stress affects the composition and fluidity of the plasma membrane, which in turn regulates the dissociation of ANAC062. Accordingly, the proteolytic processing of ANAC062 under cold is triggered by cold-induced changes in membrane fluidity (Seo et al., 2010; Degenkolbe et al., 2012). The nuclear-localized transcription factor ANAC103 and the processed form of ANAC062 bind the promoter region of UPR downstream genes as BiP, calnexin, reticulin, and PDI (Sun et al., 2013; Yang et al., 2014a). Although the activation mechanism of bZIP60/HAC1/XBP1 is conserved among eukaryotic cells, plant cells seem to have evolved new specific transmembrane components, such as ANAC062/ANAC103, to strengthen and perhaps amplify the pro-survival function of plant UPR.

## Conserved Features of the ER Stress-Induced Cell Death in Plants

Severe and persistent ER stress jeopardizes cell stability either due to an excess of unfolded proteins or Ca^2+^ imbalance, thereby, PCD is activated to maintain the integrality of the whole system (Jäger et al., 2012). Although ER-induced cell death occurs *via* apoptosis or autophagy, a crosstalk between the two pathways has been described in mammals (Maiuri et al., 2007). Autophagy can inhibit cysteine protease activities, including apoptosis-associated caspases, whereas apoptosis induces the degradation of autophagy-related proteins (ATG). Nevertheless, autophagy can also strengthen apoptosis processes in some cases, leading to a complex interplay of these processes upon ER stress (Song et al., 2017). In maize, autophagy responses range from pro-survival effects, reducing the oxidative stress response, to pro-death responses, by upregulating the Cep1-like cysteine protease (Srivastava et al., 2018).

In mammalian cells, the transmembrane mammalian ER sensors IRE1, ATF6, and PERK, in addition to functioning in ER homeostasis recovery, in critical cases, activate PCD (Nirmala and Lopus, 2020). However, the mechanisms coordinating pro-survival or apoptotic signaling have yet to be fully elucidated. In addition to inducing pro-survival related genes (Cross et al., 2012), IRE1 signaling also activates the apoptotic signaling kinase 1 and, in a tumor necrosis factor receptor-associated factor 2-dependent manner, it activates the Jun-N-terminal kinase (JNK), the main protein of a pathway described to be apoptotic in late ER stress responses but antiapoptotic in earlier responses. The IRE1-mediated JNK activation acts upstream of XBP splicing (Urano et al., 2000; Brown et al., 2016). The RIDD activity selectively degrades mRNA encoding foldases; thereby, prolonged activation of RIDD signaling promotes cell death (Han et al., 2009; Hollien et al., 2009).

In Arabidopsis, IRE1a and IRE1b isoforms are localized in the perinuclear ER, and concomitant with bZIP60 splicing, IRE1 exhibits degradation RIDD activity on another ribosome-associated mRNA in the ER (Li and Howell, 2021). Tunicamycin-induced RIDD activity of ZmIRE1 leads to the downregulation of various peroxidase genes in the early phase of stress; however, along with other ZmIRE1 antiapoptotic activities, the RIDD activity is attenuated during the late phase. There are several proposed mechanisms of plant IRE1 attenuation, including the formation of an ERdJ4/IRE1/BiP complex (Amin-Wetzel et al., 2017; Srivastava et al., 2018).

Like XBP-1, the Golgi-matured ATF6 activates the expression of UPR genes during mild stress and, in case of persistent stress, upregulates PCD genes, including the pro-death bZIP transcription factor CHOP (Yang et al., 2020). In Arabidopsis, although the activation mechanism of the transcription factors bZIP17 and bZIP28 under UPR shares conservation with the ER-Golgi traffic-mediated activation of ATF6, the role of the plant transcriptional factors during PCD has not been fully uncovered (Sanchez et al., 2000; Eichmann and Schäfer, 2012). The endoplasmic reticulum (ER)-resident transmembrane protein Bax inhibitor-1 (BI-1) is a cell death regulator in plants (Sanchez et al., 2000), which has been recently shown to modulate ER stress-induced PCD by attenuating the pro-survival function of bZIP28 during ER stress recovery (Ruberti et al., 2018). BI-1 acts in parallel to the UPR pathway to modulate ER stress-mediated PCD in Arabidopsis (Watanabe and Lam, 2008). The BI-1-mediated cell death regulation is activated by physical interactions with key modulators of Ca^+2^ signaling and lipid metabolism (Ishikawa et al., 2011; Nagano et al., 2019). Despite the BI-1 pro-survival role, new studies have been shown that BI-1 can interact with ATG6 to induce autophagy and PCD (Xu et al., 2017). Interestingly, in Arabidopsis, plant BI1 antagonizes bZIP28 function, and unlike mammalian BI1, it does not suppress the IRE1-ribonuclease activity demonstrating unique features in the modulation of the UPR signaling-mediated PCD modules (Ruberti et al., 2018). The third mammalian ER sensor, the transmembrane protein kinase (PERK), which shutdowns global translation by phosphorylating elf2α, has not been identified in plants. Nonetheless, under cold stress, the proteins AtGCN1 and AtGCN2 are involved in the plant elf2α phosphorylation (Wang et al., 2017).

As the central calcium storage organelle, the ER has calcium-dependent resident foldases, making a Ca^2+^ balance critical for protein synthesis homeostasis. High levels of calcium release can also lead to the accumulation of the cation in the mitochondria, leading to oxidative stress and hence activation of PCD (Marchi et al., 2018). Therefore, the cell also has calcium regulators like Bcl-2, a calcium sensor that modulates its release from the ER and regulates mammalian apoptosis (Pinton and Rizzuto, 2006). Interestingly, plants do not have Bcl-2 at a DNA level, but mammalian Bcl-2 and other homologs conserve their function when expressed in plants (Dickman et al., 2001). The ability of heterologous Bcl-2 in protecting the plant cell from death during severe biotic and abiotic stresses suggests conservation with a putative plant Bcl-2 at a structural level (Williams et al., 2014).

The Bcl-2-associated athanogene (BAG) family is another example of conservation beyond the sequence level in plants (Williams et al., 2014). BAG proteins are a multifunctional group of cochaperones with diverse subcellular locations (Thanthrige et al., 2020). An interaction screening for Bcl-2 partners identified the first BAG protein (Takayama et al., 1995). BAG1 is a cytoprotective protein that activates Bcl-2 to protect mitochondrial integrity by Ca^2+^ sensing, permeability, and regulation. The protein also has a conserved-Hsp70 binding domain that activates the chaperone leading to the inhibition of apoptosome formation (Planchamp et al., 2008). Although BAG family homologs have not been identified in the Arabidopsis genome *via* multiple sequence alignment, more robust structural comparison methods have uncovered seven putative plant BAGs (Doukhanina et al., 2006). Four of the seven BAG family members have domain organization similar to the mammalian counterparts, whereas three copies in the Arabidopsis genome possess a divergent calmodulin-binding domain (Li and Dickman, 2016). The endoplasmic reticulum resident BAG7 has been shown to play a central regulatory role in the UPR pathway under ER, cold, and heat stress (Williams et al., 2010). Furthermore, the hypersensitivity phenotype of BAG7 mutants to autophagy inducers indicates that BAG7 may regulate autophagy pathways (Williams et al., 2010). BAG7 binds to the Hsp70 paralog BiP2, a molecular marker of UPR and one of the negative regulators of the plant-specific NRP-mediated cell death pathway (Williams et al., 2010; Reis et al., 2016). In normal conditions, the cell death suppressor BAG7 binds bZIP28 and BiP2 in the ER. Under ER stress, BAG7 is sumoylated and dissociates with bZIP28 from BiP2, and both are proteolytically processed to relocate to the nucleus. In the nucleus, sumoylated BAG7 interacts with WRK29 to induce the expression of BAG7 and other UPR genes (Li et al., 2017). Therefore, plant BAGs retain several biochemical properties reminiscent of mammalian BAGs that suggest similar inhibitory roles in cell death events.

## ER Stress-Induced Plant-Specific Cell Death Signaling: Mechanisms and Regulation

**Figure 3:**
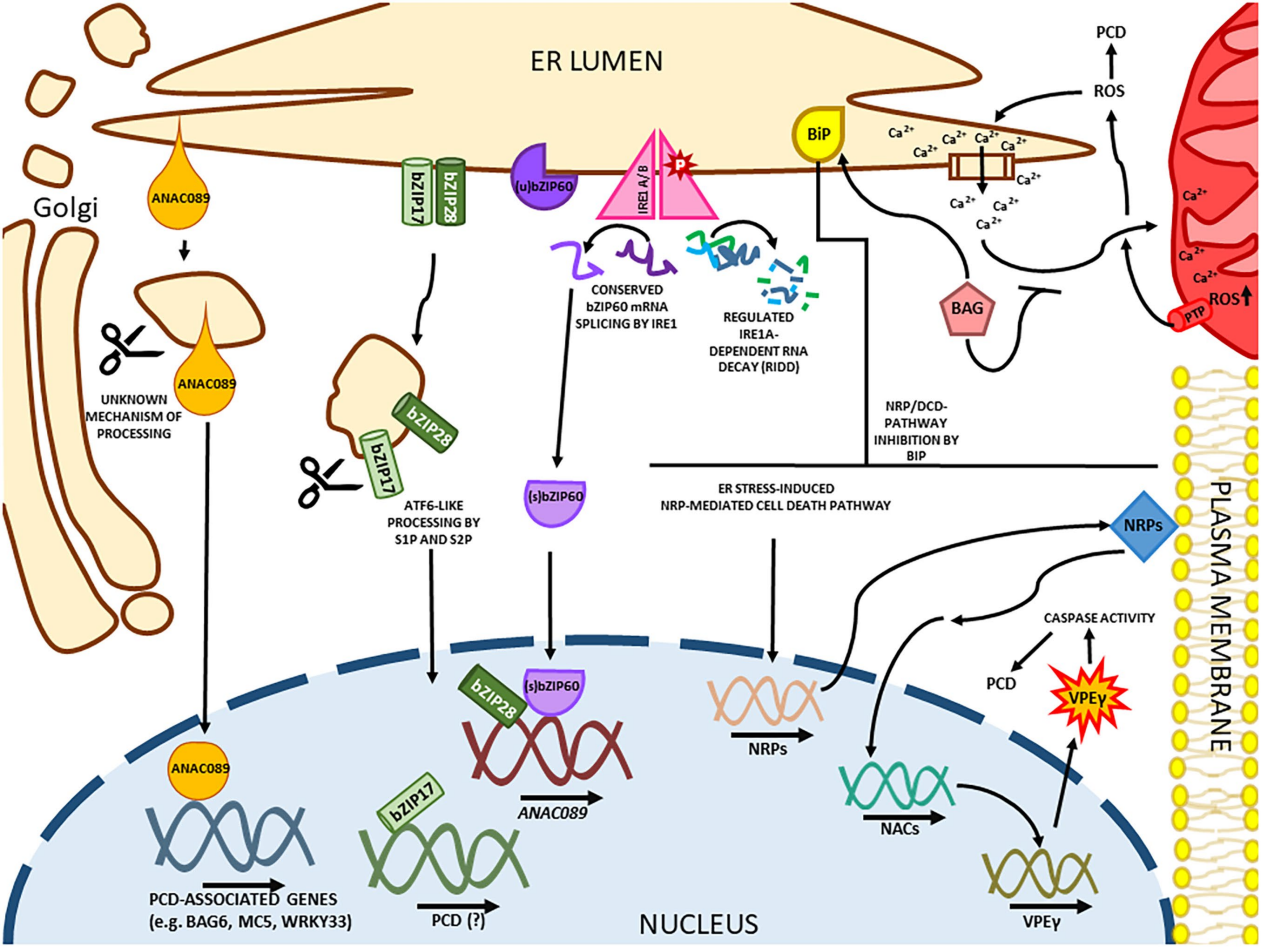
ER stress-induced cell death in plants. In a mammalian-conserved mechanism, IRE1 is responsible for the splicing of bZIP60, which loses its transmembrane domain causing a relocation to the nucleus. IRE1A has an unspecific ribonuclease function during cell death, promoting the Regulated IRE1a-dependent RNA Decay. Like the ATF6 factor in mammals, the transmembrane ER sensors bZIP17 and bZIP28 are processed in the Golgi apparatus. Both bZIP28 and bZIP60 matured transcription factors have pro-survival functions in the nucleus but also upregulate pro-apoptotic genes like ANAC089 in Arabidopsis. ANAC089 is a plant-specific NAC-containing factor that is processed in the Golgi in a S1P/S2P-independent mechanism. Without its transmembrane domain, ANAC089 upregulates programmed cell death (PCD)-associated genes. Another plant-specific arm of ER-induced PCD is the developmental cell death (DCD) domain-containing asparagine-rich protein (NRP)-mediated cell death response, where the NRP genes are upregulated and induce the expression of ANAC036 in Arabidopsis, culminating in the expression of the cell death executor gammaVPE. The NRP/DCD pathway is attenuated by the expression of the molecular chaperone BiP. Prolonged ER stress also promotes the Calcium release from the organelle and accumulation in the mitochondria. The cation accumulation in the mitochondria leads to a ROS burst and the formation of mitochondrial PTPs. The mitochondrial ROS leakage promotes the Calcium release from the ER creating a positive feedback loop. A few BAG proteins are BiP binding partners, and this protein family acts as calcium sensors, inhibiting the ion accumulation in the mitochondria.

The plant cell can trigger pro-survival or pro-death signaling pathways to dictate the cell fate in response to ER stress. The NAC (NAM/ATAF/CUC) transcription factors constitute one of the main components of this second layer of the signaling response. As plant-specific transactivator factors, NACs are involved in plant-specific mechanisms underlying ER stress-induced cell death response, often associated with prolonged ER stress conditions (Figure 3). The NAC module of transducers and other cell death regulators are represented by: (i) the DCD/NRP-NAC-VPE (vacuolar processing enzyme) cell death signaling circuit, (ii) the (bZIP28/bZIP60)-ANAC089 cell death signaling module, and (iii) the ER–mitochondria crosstalk mediated by ANAC013/ANAC017.

The NRP-NAC-VPE cell death signaling module, also known as development and cell death domain-containing N-rich protein (DCD/NRP)-mediated cell death signaling, integrates osmotic and ER stress into a signaling cascade that leads to a cell death fate (reviewed in Fraga et al., 2021). The ER and osmotic stress stimuli induce Glycine max (Gm)ERD15 expression that activates the DCD/NRP promoters (Alves et al., 2011). The small-sized, acidic, and hydrophilic transcription factor GmERD15 (Early Dehydration Responsive) belongs to a PAM2 domain-containing protein family, first identified due to its rapid response to drought stress (Kiyosue et al., 1994). GmERD15 can recognize the palindromic sequence −511AGCAnnnnnTGCT−500 on the NRP-B promotor (Kiyosue et al., 1994; Alves et al., 2011).

The GmERD15-mediated induction of two plant-specific DCD/N-rich proteins, GmNRP-A and GmNRP-B, leads to enhanced cell death markers *in planta*, including chlorophyll loss, DNA fragmentation, caspase-3-like activity, malondialdehyde production, and leaf yellowing (Costa et al., 2008; Reis et al., 2011, 2016). This cell death response is initiated by induction of the GmNRPs, which activate a signaling cascade culminating with GmNAC030 and GmNAC081 induced expression (Faria et al., 2011; Mendes et al., 2013). These transcription factors, GmNAC081 and GmNAC030, form a heterodimer that binds to cis-regulatory sequences and activates target promoters from hydrolytic enzyme-encoding genes, including VPE, a caspase-1-like vacuolar processing enzyme, which is an effector of cell death (Mendes et al., 2013; Pimenta et al., 2016). The vacuole-localized cysteine protease (VPE) can be self-activated through a hydrolytic cleavage step and, in turn, mediates activation of vacuolar enzymes, crucial to the vacuolar collapse-mediated cell death, a plant-specific PCD event (Hara-Nishimura et al., 2005; Hatsugai et al., 2015).

First identified in soybean, the NRP-NAC-VPE module is conserved in other plant species. The Arabidopsis orthologs AtNRP1, AtNRP2, ANAC036, and γVPE induce cell death in *N. benthamiana* leaves, and their knockout lines display enhanced tolerance to ER stress and cell death (Figure 3; Reis et al., 2016; de Camargos et al., 2019). Furthermore, stress-mediated induction of ANAC036 and γVPE requires the NRP1 function (Reis et al., 2016). These interpretations have been challenged by recent studies conducted with the double mutant *nrp1/nrp2* in Arabidopsis (Yang et al., 2021). In contrast to the cell death resistant phenotype displayed by 15 days-old *nrp1* knockout seedlings (Reis et al., 2016), the *nrp1/nrp2* double mutant exhibits enhanced ER stress-induced cell death response at 7 days after germination (Yang et al., 2021). The apparent contradiction between these studies may suggest that specific pathways regulating the NRP-mediated cell death signaling cascade might operate in different stages of plant development. While the pro-survival function of NRPs culminates with inhibition of cell death-related metacaspases under ER stress conditions, the pro-death function of NRP1 has been linked to the induction of the ANAC036-VPE signaling module (Reis et al., 2016; Yang et al., 2021). More recently, a Cd^2+^-mediated cell death response was associated with induction of both ER stress and the NRPs-GmNACs-VPE signaling module in soybean at the vegetative and reproductive developmental stages (Quadros et al., 2021). Nevertheless, the ER stress-mediated activation of the NRPs-GmNACs-VPE signaling module has not been investigated in soybean seedlings or during germination, and the possibility that NRPs activate specific signaling modules under differential developmental programs remains enigmatic. The molecular chaperone BiP attenuates the NRP/DCD-mediated cell death response by modulating their components expression and activity in soybean, tobacco, and Arabidopsis (Valente et al., 2009; Reis et al., 2011).

Contradictory results have also been reported for Arabidopsis AGB1-mediated signaling events that trigger UPR-associated cell death in plants (Wang et al., 2007; Chen and Brandizzi, 2012). AGB1 is the Gβ subunit of the heterotrimeric G protein, which has been demonstrated to associate partially with the ER membrane. Inactivation of the single-copy gene *AGB1* has been shown to impair ER stress-induced cell death and attenuate the induction of UPR-specific target genes in grown plants (Wang et al., 2007). In contrast, a more recent report demonstrated that three AGB1 mutants, *agb1-1*, *agb1-2*, and *agb1-3*, displayed oversensitivity to ER stress during germination (Chen and Brandizzi, 2012). Although the underlying mechanism for AGB1-mediated cell death remains unsolved, whether AGB1 would associate with different signaling modules at different developmental stages has not been investigated.

Another plant-specific transcriptional factor from the Arabidopsis NAC family, *ANAC089*, uses a different signaling module to mediate ER stress-mediated cell death responses (Figure 3). Under severe ER stress conditions, the ER membrane-anchored transcription factor ANAC089 relocates from the ER membrane to the Golgi, where it undergoes proteolytic cleavage by a yet-to-be-identified protease (Ai et al., 2021). The C-terminal ER lumen small size tail of ANAC089 has no canonical S1P cutting site, and loss of S2P function does not impair the correct ANAC089 processing in the Golgi (Liu et al., 2007a; Yang et al., 2014b). The processed ANAC089 is redirected to the nucleus to promote the induction of caspase-like activities, the NRP-ANAC-VPE cell death module, and downstream PCD-associated genes, including BAG6 (Bcl-2-associated athanogene family member), MC5 (metacaspase 5), WRKY33 (autophagy-related gene), and aspartyl protease A39 (Yang et al., 2014b). Accordingly, the overexpression of truncated ANAC089 without the transmembrane domain induces PCD, whereas the ANAC089 RNAi plants display ER stress tolerance (Yang et al., 2014b). Under ER stress conditions, ANAC089 is induced by bZIP28 and bZIP60, which bind to the UPRE-I element on the ANAC089 promoter. Besides the increase in ANAC089 protein levels, the ANAC089 proteolytically processed form is tightly controlled and only activated under severe ER stress (Yang et al., 2014b). Accordingly, tobacco mosaic virus and phytophthora infections induce ANAC089 expression, and the ER stress-induced immune signal promotes the ANAC089 relocation to the nucleus to activate genes involved in PCD (Li et al., 2018; Ai et al., 2021).

The crosstalk between organelles is essential for maintaining cellular homeostasis. The ER can cooperate with mitochondria and chloroplast through inter-organellar communication that triggers specific signaling pathways to promote cell survival or death events (Liu and Li, 2019). Ca^2+^, ROS, MEcPP, and the ER-anchored transcription factors ANAC013/ANAC017 are implicated as the core components of this inter-organellar crosstalk. ANAC013 and ANAC017 have been shown to be involved in retrograde mitochondrial regulation during stressful conditions (De Clercq et al., 2013; Ng et al., 2013). Under ER stress, ANAC017 also plays a protective role in ER stress tolerance, inducing the expression of molecular chaperones, bZIP60, and ER stress-responsive genes (Chi et al., 2017). The disruption of ER and mitochondrial homeostasis might activate unknown proteases to process the two ER-anchored NAC proteins and hence relocate them from the ER membrane to the nucleus. Together, the ER-anchored ANAC013 and ANAC017 integrate the mitochondria and chloroplast ROS signaling through interaction with a nuclear protein Radical-induced Cell Death1 (Shapiguzov et al., 2019).

The ER serves as intracellular storage of Ca^2+^, and the ER lumen concentration of Ca^2+^ is vital to facilitate the activity of ER chaperones and foldases (Berridge, 2002). Mitochondria and ER can communicate through Ca^2+^ mobilization. Furthermore, perturbation in ER functions promotes the release of Ca^2+^ to mitochondria, resulting in a mitochondrial permeability transition pore (PTP), which triggers a cell death signaling pathway that intensifies ER Ca^2+^ release by a positive feedback loop, decreasing ER protein-folding capacity (Williams et al., 2014). In addition to chloroplast and mitochondria, ROS also can be produced in the ER lumen to provide an oxidizing environment for PDI disulfide bond formation (Ozgur et al., 2015). The H_2_O_2_ permeability of the ER membrane allows the ER to influence mitochondrial ROS production, and the mitochondrial ROS can induce ER Ca^2+^ mobilization and expression of UPR components (Ramming et al., 2014; Ozgur et al., 2018). Furthermore, the plastidial metabolite MEcPP has been shown to directly trigger the induction of selected UPR genes, coupling the chloroplast and ER homeostasis (Walley et al., 2015).

## ER Stress-Mediated Autophagy

Occasionally, the efforts of the UPR pathway and the ERQC and ERAD systems under extreme circumstances cannot restore the ER balance. In this case, the ER stress might lead to autophagy, cell death, or even the death of the whole plant (Wan and Jiang, 2016). Autophagy (meaning “self-eating”), conserved in all eukaryotes, is a cell-sparing process and represents a macromolecule degradation process, in which cells recycle cytoplasmic contents, whole or pieces of organelles through the lysosomes in metazoans, or the vacuole in yeast and plants (Liu and Bassham, 2012; Wan and Jiang, 2016). Under optimal growth conditions, this process is kept at basal levels but is highly upregulated by a wide variety of biotic and abiotic stresses, such as nutrient starvation, pathogen infection, heat, or drought stress (Liu et al., 2009; Li et al., 2015; Gomez et al., 2021). Autophagy may also be triggered by ER stress through ER stress inducers, such as TM and DTT (Liu et al., 2012), leading to engulfment of ER membranes (ER-phagy; Grumati et al., 2018). Macromolecules resulting from autophagic degradation are reused by the cell to reestablish basal metabolism and stimulate the plant’s acclimatization and resistance to adverse environmental conditions (Minina et al., 2018). Three main types of autophagy have been described based on their mechanisms and membrane dynamics: macroautophagy (Yang and Klionsky, 2010), microautophagy (Oku and Sakai, 2018), and autophagy mediated by direct target translocation across the lysosomal membrane, such as chaperone-mediated autophagy (Orenstein and Cuervo, 2010). In plants, macroautophagy and microautophagy have already been described (Bassham, 2007).

Autophagy is a self-destructive process that engulfs non-essential or damaged cellular components, including organelles, in characteristic double-membrane vesicles known as autophagosomes with subsequent cargo delivery to the vacuole where they are degraded or recycled. Into the vacuole, the outer membrane of the autophagosome fuses with the tonoplast, and the inner membrane with the cargo is degraded by vacuolar hydrolases (Pu and Bassham, 2013). A set of autophagy-related genes (ATG) and proteins is essential for this process (Mizushima et al., 2011). In yeast, more than 30 ATG genes have been identified, and many of them are also present in mammals and plants (Mizushima et al., 2011; Liu and Bassham, 2012). These genes can be divided into several functional groups (Yang and Klionsky, 2010; Bao and Bassham, 2020): the ATG1-ATG13 complex, which senses the signal and initiates autophagosome formation through ATG9 recruitment; ATG9 and associated proteins that acquire lipids for the expansion of the phagophore (a cup-shaped double-membrane that expands to form an autophagosome); the phosphoinositide 3-kinase complex, which, together with ATG9, is required for the initiation of autophagosome formation; and two ubiquitin-like conjugation systems, ATG5-ATG12/ATG16 and ATG8-phosphatidylethanolamine (PE). The first system acts as an E3 ligase and mediates the covalent conjugation of ATG8 to a PE. The second one is required to complete autophagosome formation and cargo selection. Two types of markers have been frequently used to monitor autophagosomes in plants: green fluorescent protein-ATG8 fusion proteins, because the ATG8 essential role in autophagosome formation and its stable localization in both sides of autophagosome membranes (Zeng et al., 2019); and monodansylcadaverine staining, an acidotropic dye that stains acidic membrane compartments (Contento et al., 2005).

As mentioned before, upon continuous ER stress, ER-phagy is triggered to degrade some of the misfolded/unfolded proteins accumulated in the ER (Grumati et al., 2018). In yeast and plants, autophagy is closely associated with the ER, which provides autophagosome membranes and is a target for autophagy during ER stress (Marshall and Vierstra, 2018; Zeng et al., 2019). In ER-phagy, specific cargo receptors are needed to interact with both ATG8 and the target for degradation. The yeast ER-phagy pathway requires the receptors ATG39 and ATG40, two ER membrane proteins that load the ER subdomains into autophagosomes (Mochida et al., 2015). In mammals, the ER-phagy receptors, including FAM134B, RTN3, ATL3, TEX264, CCPG1, and Sec62, were identified through an inefficient proteasomal function of ERAD under the UPR (Khaminets et al., 2015; Fumagalli et al., 2016; Grumati et al., 2017; Smith et al., 2018; An et al., 2019; Chen et al., 2019;). The first plant ER-phagy receptors reported were the *A. thaliana* ATG8-interacting proteins ATI1 and ATI2 (Honig et al., 2012). Localized in the ER membrane under normal conditions, these single transmembrane domain receptors possess an ATG8 interacting motif and do not have homologs in yeast and mammals. Under carbon starvation, ATI1 and ATI2 are transported to the vacuole upon interaction with ATG8 (Honig et al., 2012). Although plant receptors for autophagy are largely unknown, some homologs of mammalian receptors are encoded by the plant genomes, including Lnp1, calnexin, ATL3, and Sec62 (Zeng et al., 2019). Whether they have a similar function in autophagy remains obscure. For instance, Arabidopsis SEC62 is an ER transmembrane protein that co-localizes with ATG8 in autophagosomes, and the atsec62 mutant is hypersensitive to ER stress, while overexpression of AtSEC62 confers ER stress tolerance (Hu et al., 2020). Thus, AtSEC62 might be an ER-phagy receptor in plants. Despite the lack of knowledge of these receptors, IRE1 has been proved indispensable for plant ER stress-induced ER-phagy.

IRE1 is generally involved in the ER stress-induced autophagy, although it is differently regulated in yeast (Bernales et al., 2006), animals (Ogata et al., 2006), and plants (Liu et al., 2012). In yeast, the biosynthesis of ATG8p, an essential component of autophagosome formation, relies on the splicing of HAC1 by IRE1 (Yorimitsu et al., 2006) and, hence, yeast autophagy is dependent on the RNase function of IRE1. Differently, mammals require the kinase function of IRE1 as the c-JNK pathway responsible for triggering autophagy depends on IRE1 activity (Urano et al., 2000; Ogata et al., 2006). Unlike yeast, autophagy in plants is also dependent on the IRE1 RNase function; however, the splicing of bZIP60, homologous to HAC1, is irrelevant to the process. More specifically, IRE1b, but not IRE1a, has been shown to be required for ER stress-induced autophagy (Liu et al., 2012). Both Arabidopsis *ire1a* and *ire1b* null mutants display similar expression profiles of autophagy-related genes and similar levels of autophagosome formation as wild-type plants under nutrient deficiency conditions. However, the autophagosome formation is abolished under treatment with the ER stress inducers TUN and DTT in ire1b plants, but not in ire1a knockout lines (Liu et al., 2012). Other studies have shown that the RIDD function of AtIRE1b, but not its protein kinase activity or splicing target bZIP60, is responsible for regulating this event (Bao et al., 2018). Furthermore, 3 out of 12 RIDD targets potentially repress autophagy during normal conditions, while during ER stress, they are degraded to release this repression. Although IRE1b is not necessarily the direct elicitor of autophagy, it may promote the RNA degradation of transcription factors that interfere with the induction of autophagy (Bao et al., 2018). Nevertheless, further investigations are needed better to understand ER stress-induced autophagy and its components in plants.

## ER Stress-Induced PCD in Plant Immunity

ER function is associated with plant innate immunity on several levels. ER plays an essential role in processing antimicrobial proteins delivered to the site of the microbial attack by the secretory pathway *via* vesicle-mediated transport. Non-expressor of PR genes 1 (NPR1) coordinately controls the upregulation of PR genes and genes encoding proteins of the secretory pathway during salicylic acid (SA)-dependent systemic acquired resistance (SAR). Exogenous SA induces the processing of bZIP28 and splicing of bZIP60 in *Arabidopsis* and rice, linking the IRE1 activation to defense responses, which may represent a second branch regulating SA-dependent ER marker genes independently of NPR1 (Nagashima et al., 2014). A more recent report has demonstrated that an SA-independent ER stress-induced redox may promote the translocation of NPR1 to the nucleus, where it suppresses the transcriptional role of bZIP60 and bZIP28 in the UPR (Lai et al., 2018).

Consistent with the interpretation that IRE1 may be a positive regulator of SA-mediated defense responses in *Arabidopsis*, *ire1a* and *bzip60* mutants display enhanced susceptibility to the hemibiotrophic pathogen *Pseudomonas syringae* and attenuated SAR (Moreno et al., 2012). Likewise, the UPR branch IRE1/bZIP60 plays an essential role in turnip mosaic virus (TuMV; genus Potyvirus) and plantago asiatica mosaic virus (PlAMV; genus Potexvirus) infection (Zhang et al., 2015; Gaguancela et al., 2016). Viral pathogenesis is enhanced in the *bzip60-2* mutant and *the ire1a/ire1b* double mutant, consistent with the induced bZIP60 splicing in response to TuMV and PlAMV infection. The potyvirus membrane-binding protein 6K2 and potexvirus triple gene block 3 are the effectors that induce the IRE1/bZIP60 pathway. More recently, the bZIP17/28 branch of UPR has also been shown to be activated upon potyvirus and potexvirus infection in *Arabidopsis* (Gayral et al., 2020) and in response to rice streak virus (RSV) infection in *N. benthamiana* (Li et al., 2021). Expression of the membrane-associated viral effectors NSvc2 and NSvc4 induces the proteolytic cleavage of bbZIP17/28 and the expression of UPR-related genes. Silencing NbbZIP17/28 significantly inhibited RSV infection. Likewise, the plant susceptibility factor Resistance *to Phytophthora parasitica 1* (RTP1) has been recently shown to be involved in ER stress sensing (Qiang et al., 2021). RTP1 negatively modulates the IRE1/bZIP60 splicing activity and binds to bZIP28. In response to *P*. *parasitica* infection, *rtp1bzip60* and *rtp1bzip28* mutant plants display decreased resistance, along with attenuated induction of ER stress-responsive immune genes, suggesting that rtp1-mediated resistance to *P*. *parasitica* is coordinately regulated with UPR. Collectively these results indicated that both UPR signaling branches are linked to immune responses and provide some insights into the mechanisms by which UPR signaling cascades are coordinated with immunity.

Additionally, ER monitors the synthesis and controls the quality of several immune receptors. Specific components of ERQC mediate the processing of the pattern recognition receptors (PRR), Elongation-factor Tu (EF-Tu) receptor, which undergoes pathogen-associated molecular pattern (PAMP)-induced oligomerization with coreceptors to activate PAMP-triggered immunity (Li et al., 2009; Nekrasov et al., 2009; Saijo et al., 2009). Additional examples of plasma membrane immune receptors, which depend on ERQC for proper function, include glycosylated *Cf* proteins, linked to race-specific resistance to the fungal pathogen Cladosporium fulvum (Liebrand et al., 2012), the rice PRR XA21 involved in resistance to *Xanthomonas oryzae* pv. oryzae (Park et al., 2010), and the induced receptor kinase, implicated in N-mediated resistance of tobacco to tobacco mosaic virus (Caplan et al., 2009).

While ERQC loss-of-function mutants display enhanced susceptibility to ER stress inducers and pathogens (Wang et al., 2005; Li et al., 2009; Lu et al., 2009; Nekrasov et al., 2009; Saijo et al., 2009), the inactivation of ER-QC components enhances colonization of the mutualistic fungus *Piriformospora indica* in Arabidopsis roots (Qiang et al., 2012). The improved growth of *P*. *indica* displayed by ERQC mutants occurs only during cell death-dependent but not biotrophic colonization. *P*. *indica* activates an ER-PCD, associated with enhanced VPE/caspase 1-like activities and vacuole collapse-mediated PCD. Loss-of-function VPE mutants confirmed that the fungus depends on the VPE-mediated ER-PCD to colonize Arabidopsis roots successfully. In contrast, VPE activity has been associated with enhanced resistance to bacterial pathogens (Carvalho et al., 2014). VPE also mediates vacuolar collapse and execution of virus-induced cell death (hypersensitive response) in *Nicotiana tabacum*, which restricts virus spread to the site of infection (Hatsugai et al., 2004). Therefore, as an executioner of PCD, VPE may function dually as a susceptibility or resistance factor depending on whether the pathogen benefits from cell death or is restricted by PCD.

A recently characterized mechanism underlying ER stress-triggered PCD in immunity relies on activating the ER stress-induced membrane-anchored TF NAC089 (Yang et al., 2014b; Ai et al., 2021). In response to PAMPs from *Phytophthora capsica* and *P. syringae*, NAC089 translocates from the ER membrane to the nucleus *via* a proteolytic cleavage in the Golgi. Inside the nucleus, truncated NAC089 activates PCD-related genes (BAG6, MC5, WRKY33, aspartyl protease A39, VPE) to assemble cell death programs and restrict pathogen infection. Therefore, as an ER stress immunity regulator, NAC089 positively controls host resistance against the oomycete pathogen *P*. *capsica* and the bacterial pathogen *P. syringae*.

## Conclusion

The endoplasmic reticulum is an essential component of the cellular organism and is vital for synthesizing, folding, and quality control of proteins, lipid biosynthesis, and calcium storage. In plants, diverse abiotic stressors and biotic agents can disturb the ER operation and homeostasis, leading to ER stress conditions. The exact mechanism by which each kind of stress promotes ER stress is not known, but it is conceptually accepted that they can interfere with the ER function in some way related to protein folding. Further investigations are needed to prove this point. In response to ER stress, the plant cell can trigger pro-survival or pro-cell death pathways to restore correct cell function. Many cell strategies to alleviate ER stress have been described, including the induction of ERQC and ERAD system, UPR pathway, and under situations of prolonged stress, autophagy, and cell death signaling. However, the mechanisms underlying the coordination of recovery or death responses are still largely undescribed.

The plant UPR are transduced by a bipartite signaling module, involving the ER membrane-anchored stress sensors bZIP17/28 and IRE1 (through specific splicing of bZIP60), which are responsible for upregulating ER-resident chaperones and stress-responsive genes. Despite all knowledge about this signaling pathway, there are still some missing details regarding the molecular mechanisms of UPR in plants. For instance, we still do not know the exact protease responsible for the first cleavage of bZIP28 in its transmembrane domain, and the mechanisms of the IRE1 stress-sensing process remain to be elucidated. Other topics worth investigating involve the function of bZIP60u and IRE1c under normal and stressful situations and the role of plant transcriptional factors during PCD.

If these cytoprotective pathways cannot stabilize and alleviate the ER stress, autophagy or PCD may occur. Autophagy is a self-destructive but cell-sparing process that, although described in plant cells, some components have yet to be identified. For example, what are the plant receptors for autophagy? If IRE1b is not necessarily the direct elicitor that promotes autophagy, what induces autophagy in response to stress? Important to mention that, although autophagy is considered a pro-survival mechanism, it can also strengthen apoptosis processes in some cases, raising the question of how the decision of life-to-death is taken.

Similarly, NRPs, the upstream components of a plant-specific ER stress-induced cell death signaling, have been shown to display both pro-death and pro-survival activities. The possibility that NRPs activate specific signaling modules under different developmental stages needs to be investigated. Not less importantly, some orthologs of the plant-specific NRP-mediated cell death pathway still need to be identified in Arabidopsis and other plant species.

A relevant question arises from all these signaling profiles. What situations and circumstances determine the turning point at which the cell switch on the pro-survival profile to pro-death modules? Addressing these questions is needed for a better understanding of the plant physiological response to ER stresses, such that this knowledge can be applied for genetically engineering superior crops.

## Author Contributions

ES, OF, and CO wrote the drafts. EF and PR conceived and supervised the review topics. All authors contributed to the article and approved the submitted version.

## Funding

This work was partially funded by CAPES finance code 001, CNPq, FAPEMIG, and the National Institute of Science and Technology in Plant-Pest interactions. ES and CO are recipients of a CAPES graduate fellowship, and OF is supported by a FAPMIG graduate fellowship.

## Conflict of Interest

The authors declare that the research was conducted in the absence of any commercial or financial relationships that could be construed as a potential conflict of interest.

## Publisher’s Note

All claims expressed in this article are solely those of the authors and do not necessarily represent those of their affiliated organizations, or those of the publisher, the editors and the reviewers. Any product that may be evaluated in this article, or claim that may be made by its manufacturer, is not guaranteed or endorsed by the publisher.
